# Ambulatory Pathway for the Management of Superficial Abscesses: Criteria for Safe Provision of Care

**DOI:** 10.7759/cureus.29545

**Published:** 2022-09-24

**Authors:** Mojolaoluwa Olugbemi, Ayesha Ahmed, Aliya Prihartadi, Lucy Ridgway, Thomas Athisayaraj, Boby Sebastian

**Affiliations:** 1 General Surgery/Colorectal Surgery, West Suffolk Hospital, Bury St Edmunds, GBR; 2 General Surgery, West Suffolk Hospital, Bury St Edmunds, GBR; 3 Internal Medicine, West Suffolk Hospital, Bury St Edmunds, GBR; 4 Colorectal Surgery, West Suffolk Hospital, Bury St Edmunds, GBR

**Keywords:** pathway care model, incision and drainage of abscess, day case surgery, ambulatory care, superficial abscess

## Abstract

Background

Superficial abscesses are common surgical emergencies and unless complicated, are considered a low-priority emergency often leading to delayed primary treatment. Patients who are clinically stable benefit more from an ambulatory-care approach to their treatment.

Aim of study

This is a retrospective study to investigate the current practice of treatment of superficial abscesses at West Suffolk Hospital, United Kingdom, and evaluate the potential impact of implementing care via the ambulatory pathway to improve patients' experience, optimise the use of hospital resources and identify areas for service improvement.

Patients and methods

A total of 76 patients who required inpatient care for drainage of their superficial abscess under general anaesthesia over six months were a part of the study. Admission, procedure, and hospital stay characteristics were evaluated as well as our proposed superficial abscess ambulatory-care pathway (SAAP) criteria.

Results

The mean age was 39 ± 18 years. Perianal and pilonidal abscesses accounted for 53% of the procedures. Only 24% of the patients had a C-reactive protein (CRP) >100 at admission. The average waiting time before intervention was 19 hours (± 16.25) with patients spending two days on average in the hospital for a procedure that lasted an average of 31 minutes. Overall, 39.5% of the patients received drainage of their abscess on the same day of presentation to the hospital with only five discharges on the same day of admission. Of the total patients, 52.6% met our SAAP criteria for ambulatory care and could have avoided inpatient care and benefitted from same-day discharges.

Conclusion

Ambulatory care of a superficial abscess is a safe, feasible approach. We recommend establishing an ambulatory care pathway for the management of superficial abscess cases with our SAAP criteria serving as a useful objective guide for effective and safe triage of patients with reduced hospital stay and more efficient utilisation of resources.

## Introduction

The incision and drainage of an abscess is a quick, primary operative intervention to treat an abscess [1]. Superficial abscesses form a significant portion of surgical emergencies and ideally, same-day incision and drainage should be provided [2]. However, unless complicated, they are often considered a low-priority emergency procedure often leading to delays in treatment required to alleviate symptoms [1,3]. The Association of Anaesthetists and the British Association of Day Surgery guidelines support the utilisation of semi-elective emergency pathways to provide certain acute interventions, which, though urgent, are at risk of deferment due to the provision of services that take precedence. Clinical conditions in which operative intervention can be safely postponed for up to 24 hours, including incision and drainage of abscess, are recommended for service provision in this fashion in the guideline [4]. In the United Kingdom (UK), there is no nationally defined pathway to provide a service meeting these characteristics despite long-established patient and hospital benefits inherent in ambulatory-care pathways; therefore, care approaches vary widely amongst different hospitals [5]. The aim of this study was to investigate the current management approach of superficial abscesses at West Suffolk Hospital, Bury St Edmunds, England, UK, and evaluate the potential impact of implementing care via the ambulatory pathway to improve patients' experience, optimise the use of hospital resources, and identify areas for service improvement.

This article was previously presented as a meeting abstract at the 2022 Association of Surgeons of Great Britain and Ireland (ASGBI), May 3-5, 2022, and the abstract has been published in the British Journal of Surgery, Volume 109, August 2022 (doi.org/10.1093/bjs/znac245.170)

## Materials and methods

This is a retrospective study of the care practices for patients requiring hospital admission and inpatient care for the drainage of superficial abscesses by the general surgery team under general anaesthesia. We included adult patients (>16years) treated over a six-month period between May and November 2021 at West Suffolk Hospital, Bury St Edmunds, England, UK. Relevant data were collected from admission clerking notes, daily entries, operation notes, anaesthetic charts, laboratory results, and discharge summaries. Analysis of the time at admission, admission clinical observations, laboratory results on admission, patient’s co-morbidities, waiting period from admission to surgery, the type of procedure performed, the duration of operation including anaesthesia, and the total length of inpatient stay was carried out. We designed an ambulatory care pathway capable of ensuring safe and efficient operative management of superficial abscesses (Superficial Abscess Ambulatory-care Pathway (SAAP)). Furthermore, using the data derived as well as clinical experience, we determined criteria for identifying patients whose treatment could have been safely delayed for 24 hours based on objective clinical and laboratory parameters (Table 1). During our sub-group analysis, we identified the patients in our total population who met all the criteria and analysed their data.

**Table 1:** SAAP criteria SAAP: superficial abscess ambulatory-care pathway; CRP: C-reactive protein

Criteria for the SAAP (All four parameters should be present for the patient to be safely treated via this pathway)
Apyrexial on admission
White cell count < 13.5x10*9/L
CRP < 100mg/L
No significant co-morbidities/other medical concerns requiring inpatient care (No diabetes mellitus, no suspicion for necrotizing fasciitis or deep abscess, no immunosuppression)

**Figure 1:**
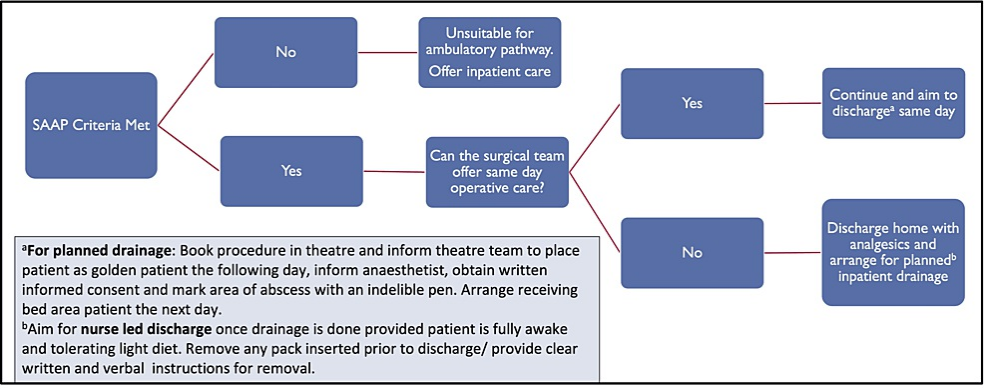
Superficial abscess ambulatory-care pathway (SAAP)

Data analysis was performed using IBM SPSS Statistics for Windows, Version 21.0 (Released 2012; IBM Corp., Armonk, New York, United States) and presented using charts and tables. Patients who met the set SAAP criteria were considered favourable for treatment in the ambulatory care pathway (Figure 1), which would allow discharge home at presentation to the emergency department and planned hospital attendance on the next morning for incision and drainage.

## Results

**Figure 2:**
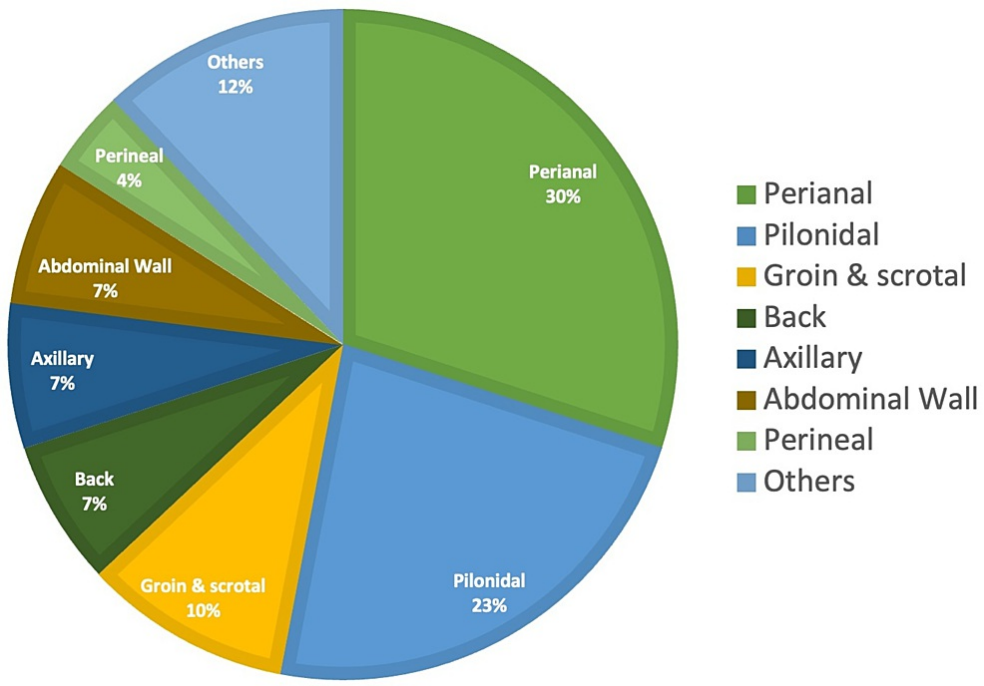
Distribution of superficial abscess sites in the study population

Seventy-six patients were admitted and managed as inpatients for superficial abscesses during the period of study. Their ages ranged from four years to 84 years (with a median of 35 years) and the mean age was 39 ± 18 years. There was male preponderance with male to female ratio of 3:2. Incision and drainage of perianal and pilonidal abscesses accounted for majority (53%) of the procedures (Figure 2).

Overall, admission blood parameters showed the mean white cell count (WCC) was 13.2x10*9/L, the mean C-reactive protein (CRP) in the total population was 58mg/L ± 71 with only 24% of the patients having a CRP >100mg/L and 36% of the patients having a WCC >13.5 x10*9/L. (Table 2). The time interval between admission and surgical intervention ranged from two hours to 94 hours (mean 19 ± 16.25 hours) while the total length of admission ranged from 7 hours to 167 hours with patients averagely spending 43 ± 32 hours in the hospital during their care and hospital bed/day utilisation during their treatment ranged from one to six days. A total of 130 beds were occupied overnight and of these, while 53 bed nights were spent awaiting surgery, 77 were spent awaiting discharge following surgery. Duration of the procedure was defined as duration from start to stop of anaesthesia. It ranged from eight minutes to 69 minutes with an average duration of 31 ± 14 minutes. Only five out of 76 patients were discharged on the day of admission.

**Table 2:** C-reactive protein, white cell count, and pyrexia in the study population

C-Reactive Protein (mg/L)	White Cell Count (10*9/L)	Pyrexia
1-50: 62%	≤13.5: 64%	Apyrexial: 86%
51-100: 14%	>13.5: 36%	Pyrexia: 14%
>100: 24%		

**Figure 3:**
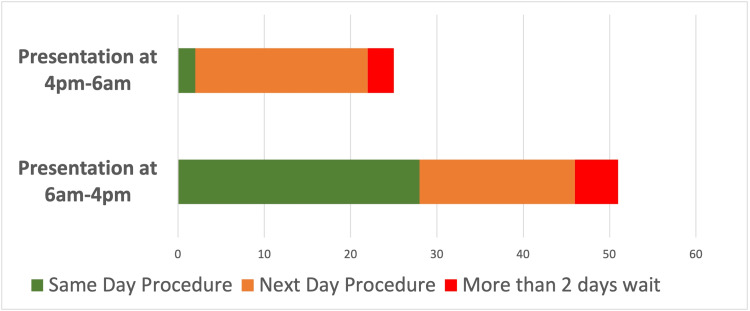
Admission to procedure timeline

The time at presentation to the emergency department was studied and patients were divided into two groups based on this. Group A presented during active working hours (6am-4pm) and Group B (>4pm to <6am) with majority (67%) of our patients presenting between 6am-4pm. Overall, 39.5% of the patients had their drainage on the same day of presentation to the hospital. While 55% of Group A patients had their procedure on the same day, only 8% of Group B patients had their procedure on the same day with majority (92%) waiting until the next day for their procedure (Figure 3). On analysis using our SAAP criteria, 40 of 76 patients included in the study met the inclusion criteria and could have qualified for ambulatory care.

## Discussion

A superficial abscess is a painful clinical condition readily treated by simple incision and drainage procedures under general or local anaesthesia depending on the preferences of the patient and surgeon [1]. This study focused on patients who required general anaesthesia for their procedure as this is the group of patients most at risk of prolonged inpatient stay for a minor procedure.

In this study, perianal abscess accounted for the majority of abscesses followed by pilonidal abscesses, both of which accounted for about a quarter each of the types of abscesses treated. Nixon et al., in another UK study, found pilonidal abscess was the most common cause in their study [6]. Pyrexia, WCC, and CRP level serve as readily available objective parameters for assessing the severity of the infection and provide clinicians with guidance during the care of patients presenting with an infection [7,8] Majority of the patients in this study did not demonstrate signs of severe infection and were well enough for a deferment of their treatment till the next day. Patients who met the set criteria could have been offered the option of receiving ambulatory care. This would have allowed discharge home at presentation to the hospital and planned hospital attendance on the next morning for incision and drainage. Ambulatory care uniquely conserves scarce hospital resources and provides a safe, effective, and more convenient care pathway for the patient with a significantly better overall patient experience than would be obtained from prolonged inpatient hospital wait for their procedure [6]. Our pathway provides clinicians with a guide to harnessing the numerous benefits to the patients and the hospital inherent in this approach to service provision.

In our study, the likelihood of a superficial abscess being drained on the same day of admission remarkably decreased when the patient presented to the emergency department after 4pm. While there was a one in two chance of same-day procedure amongst patients presenting between 6am and 4pm, the odds significantly dropped to one in 12 after 4pm. Therefore, in this latter group, patients who were well enough to be placed on an ambulatory pathway could have experienced more effective care. Reduction in the duration of inpatient stay is known to increase the number of patients who can be treated within the National Health Service (NHS) per time [9]. In addition to improving patient experience, an ambulatory-care pathway reduces inpatient hospital stay and bed occupation without compromising patient safety [3]. The daily cost of bed occupancy in the NHS using 2016/2017 prices was estimated to be £586 for a general ward bed [10]. In this study, 60% of the total bed occupation time was spent awaiting discharge demonstrating the significant cost benefit inherent in an ambulatory care pathway [11-13]. The incision and drainage of most superficial abscesses under general anaesthesia is a quick procedure requiring about half an hour in most instances inclusive of anaesthesia time. This short duration makes it favourable for placing it first in an inpatient emergency list when a separate day-surgery emergency list is not available. Although enacting this pathway can be challenging in many hospital settings, the inestimable benefits that can be harnessed make a case for the prioritisation of the development of this service. The service environment unique to each hospital must be considered along with the relevant service end users during its development. Cost savings and improved efficiency in the utilisation of resources are recognised benefits of reduced inpatient stay [13].

An ambulatory abscess pathway is a sustainable, recognised tool for efficiently managing superficial abscesses, which can be adapted to services in an individual hospital to increase achieve its goals. [14]. Standardised verbal and written information guide patients and caregivers in ensuring well-integrated, seamless care and flow through the pathway is provided. Our SAAP criteria is a useful method of guiding clinicians in selecting patients who can be safely managed. This was a small single-centre study with its findings, therefore, dependent on the current workflow of one hospital. Although both factors serve as limitations, nevertheless, it demonstrates an identifiable need to improve clinical and service provision patterns to a well-recognised group of patients who significantly add to acute surgical emergencies and its findings can be readily adapted to existing services providing care for these patients.

## Conclusions

A significant number of superficial abscess cases who presented to our hospital could have been discharged home after initial assessment, to be brought back for a scheduled procedure the following day. This would enable more resources to be available for more patients, streamline the superficial abscess care pathway, allow for more effective patient care with improved patient clinical experience. We recommend establishing an ambulatory care pathway for the management of superficial abscess cases with our SAAP criteria serving as a useful objective guide for effective and safe triage of patients to be referred to the ambulatory care pathway.
